# Supplementary material for: Losing Dnmt3a dependent methylation in inhibitory neurons impairs neural function by a mechanism impacting Rett syndrome
Source: eLife. 2020 Mar 11;9:e52981. doi: 10.7554/eLife.52981 (PMC7065908; doi:10.7554/eLife.52981)
Supplement: Transparent reporting form [file elife-52981-transrepform.docx]

***eLife’s* transparent reporting form**

We encourage authors to provide detailed information *within their submission* to facilitate the interpretation and replication of experiments. Authors can upload supporting documentation to indicate the use of appropriate reporting guidelines for health-related research (see [EQUATOR Network](http://www.equator-network.org/%20)), life science research (see the [BioSharing Information Resource](https://biosharing.org/)), or the [ARRIVE guidelines](http://www.plosbiology.org/article/info:doi/10.1371/journal.pbio.1000412) for reporting work involving animal research. Where applicable, authors should refer to any relevant reporting standards documents in this form.

If you have any questions, please consult our Journal Policies and/or contact us: [editorial@elifesciences.org](mailto:editorial@elifesciences.org).

**Sample-size estimation**

- You should state whether an appropriate sample size was computed when the study was being designed
- You should state the statistical method of sample size computation and any required assumptions
- If no explicit power analysis was used, you should describe how you decided what sample (replicate) size (number) to use

Please outline where this information can be found within the submission (e.g., sections or figure legends), or explain why this information doesn’t apply to your submission:

For behavioral tests, the number of animals tested were based on mouse numbers needed to detect robust phenotypic differences in animals with conditional knockout of *Mecp2* in the same neuron population used in this study, but on a different genetic background (PMID: 21068835). The number of mice and statistics for all behavioral tests can be found in Supplemental File 1.

For electrophysiology, at least 3-6 mice at the same age in each group was used considering the requirement of statistical accuracy and potential failure in the preparation of slices/recordings. We collected at least 4-6 neurons per day in optimized conditions, which provided the foundation of data consistency and stability. Data were discarded when the change in the series resistance was above 20% during the course of the experiment. The number of mice/neurons recorded and statistics can be found in Supplemental File 1.

Three biological replicates were used for immunofluorescence (IF) and western blots for accurate determination of biological variation. Representative IF are shown in Figure 1- Figure Supplement 1. Statistics for western blots can be found in Supplemental File 1.

The number of replicates for RNA and methylation sequencing were based on standard practices. RNA and methylation sequencing statistics can be found in Supplemental File 2 and Supplemental File 1, respectively.

Further information can be found in the materials and methods section of this manuscript.

**Replicates**

- You should report how often each experiment was performed
- You should include a definition of biological versus technical replication
- The data obtained should be provided and sufficient information should be provided to indicate the number of independent biological and/or technical replicates
- If you encountered any outliers, you should describe how these were handled
- Criteria for exclusion/inclusion of data should be clearly stated
- High-throughput sequence data should be uploaded before submission, with a private link for reviewers provided (these are available from both GEO and ArrayExpress)

Please outline where this information can be found within the submission (e.g., sections or figure legends), or explain why this information doesn’t apply to your submission:

Behavioral assays were done using two cohorts of mice for each cKO model (*Dnmt3a* cKO and *Mecp2* cKO) and their respective control mice. Animals from both cohorts were analyzed in the final data analysis. Each mouse is considered a biological replicate. Mouse numbers can be found in Supplemental File 1.

At least 3-6 mice at the same age in each group was used in electrophysiology recordings. We collected at least 4-6 neurons per day in optimized conditions. These are considered biological replicates. The number of mice/neurons recorded can be found in Supplemental File 1.

Three biological replicates were used for analysis of Dnmt3a and MeCP2 protein levels in IF and western blot experiments. Representative IF are shown in Figure 1- Figure Supplement 1. Numbers and statistics for western blots can be found in Supplemental File 1.

For RNA and methylation sequencing 4 and 2 biological replicates were used, respectively. All samples were included in the final analysis. Information and statistics for these samples can be found in Supplemental File 2 and Supplemental File 1. Data and metadata for sequencing experiments can be found at GEO: GSE123941 (RNA sequencing) and GSE124009 (methylation sequencing).

Further information can be found in the materials and methods section of this manuscript.

**Statistical reporting**

- Statistical analysis methods should be described and justified
- Raw data should be presented in figures whenever informative to do so (typically when N per group is less than 10)
- For each experiment, you should identify the statistical tests used, exact values of N, definitions of center, methods of multiple test correction, and dispersion and precision measures (e.g., mean, median, SD, SEM, confidence intervals; and, for the major substantive results, a measure of effect size (e.g., Pearson's r, Cohen's d)
- Report exact p-values wherever possible alongside the summary statistics and 95% confidence intervals. These should be reported for all key questions and not only when the p-value is less than 0.05.

Please outline where this information can be found within the submission (e.g., sections or figure legends), or explain why this information doesn’t apply to your submission:

Exact values of N and p-value definition for star annotations in figures can be found in figure legends. All error bars are SEM.

Exact values of N, p-values and full statistical analysis can be found in Supplemental Files 1 and 2.

Further description of data analysis can be found in the materials and methods section of this manuscript.

(For large datasets, or papers with a very large number of statistical tests, you may upload a single table file with tests, Ns, etc., with reference to sections in the manuscript.)

**Group allocation**

- Indicate how samples were allocated into experimental groups (in the case of clinical studies, please specify allocation to treatment method); if randomization was used, please also state if restricted randomization was applied
- Indicate if masking was used during group allocation, data collection and/or data analysis

Please outline where this information can be found within the submission (e.g., sections or figure legends), or explain why this information doesn’t apply to your submission:

Experimenter was blinded to genotypes during behavioral data collection.

For RNA and methylation sequencing samples, 1 of each of the 3 genotypes to be compared was processed together. This was done 4 times to have 4 replicates of each genotype. RNA-seq was done in with 4 replicates and 2 samples were used for methylation sequencing.

**Additional data files (“source data”)**

- We encourage you to upload relevant additional data files, such as numerical data that are represented as a graph in a figure, or as a summary table
- Where provided, these should be in the most useful format, and they can be uploaded as “Source data” files linked to a main figure or table
- Include model definition files including the full list of parameters used
- Include code used for data analysis (e.g., R, MatLab)
- Avoid stating that data files are “available upon request”

Please indicate the figures or tables for which source data files have been provided:

Data and metadata for sequencing experiments can be found at GEO: GSE123941 (RNA sequencing) and GSE124009 (methylation sequencing). Further information for these datasets can be found in Supplemental Files 1 and 2.

These data were used for Figures 2-4 and Figure 2- Supplement 1, Figure 3- Figure supplement 1, and Figure 4- Figure Supplement 1-2.
